# Transcriptome Analysis Reveals the Effects of Dietary DHA and EPA on the Hepatic Response to Unpredictable Chronic Stress in Atlantic Salmon (*Salmo salar* L.)

**DOI:** 10.3390/ani16203249

**Published:** 2026-10-15

**Authors:** Yajie Deng, Jian Chen, Hang Su, Yupeng Zhang, Jun Ding, Jihong Liu Clarke

**Affiliations:** 1 College of Fisheries and Life Science, Dalian Ocean University, Dalian 116023, China; dengyajie0220@163.com (Y.D.); chenjian20250904@163.com (J.C.); dingjun19731119@hotmail.com (J.D.); 2 Liaoning Provincial Key Laboratory of Northern Aquatic Germplasm Resources and Genetics and Breeding, Dalian Ocean University, Dalian 116023, China; 3 Chinese-Norwegian Joint Laboratory for Development and Utilization of Marine Bioresource and Sustainable Aquaculture, Dalian Ocean University, Dalian 116023, China; 4 NIBIO— Norwegian Institute of Bioeconomy Research, 1431 Ås, Norway; yupeng.zhang@pku-iaas.edu.cn

**Keywords:** Atlantic salmon, highly unsaturated fatty acids, DHA, EPA, unpredictable chronic stress, fatty acid metabolism, oxidative stress

## Abstract

Eicosapentaenoic acid (EPA) and docosahexaenoic acid (DHA) are key long-chain omega-3 fatty acids in fish nutrition, but their differential effects on Atlantic salmon under unpredictable chronic stress (UCS) remain unclear. This study compared hepatic responses to DHA- and EPA-rich diets under unstressed and UCS conditions. Atlantic salmon post-smolts were fed a low-EPA+DHA, DHA-rich, or EPA-rich diet for 10 weeks, followed by 3 weeks of UCS. Liver transcriptomes were analyzed by RNA sequencing, and selected genes were validated by quantitative reverse transcription PCR. Growth did not differ among dietary groups. Under unstressed conditions, the DHA-rich diet induced more differentially expressed genes than the EPA-rich diet (67 vs. 29) and affected pathways related to fatty acid metabolism, autophagy, cholesterol homeostasis, and antioxidant defense. After UCS, the EPA-rich diet induced a broader hepatic transcriptional response than the DHA-rich diet (205 vs. 57 differentially expressed genes), with only 12 genes shared between the two comparisons. Both diets affected fatty acid- and steroid-biosynthesis-related pathways, while gene-level responses were diet-specific. These findings reveal distinct, stress-dependent hepatic responses to dietary DHA and EPA and may inform future optimization of their nutritional provision in Atlantic salmon production.

## Simple Summary

Atlantic salmon in aquaculture are exposed to repeated and unpredictable stress, and their nutritional needs may change under these conditions. Docosahexaenoic acid (DHA) and eicosapentaenoic acid (EPA) are two important omega-3 fatty acids in fish diets, but their different roles during chronic stress are not well understood. This study compared Atlantic salmon fed diets low in these fatty acids or enriched with either DHA or EPA, before and after a period of repeated unpredictable stress. The diets did not produce significant differences in growth, but they caused clear differences in liver gene activity. Under unstressed conditions, the DHA-rich diet produced broader changes in genes involved in fat metabolism and protection against cell damage. After chronic stress, the EPA-rich diet produced broader but different changes, including genes related to fat production, immune responses, and cellular stress. These findings show that the effects of these two omega-3 fatty acids depend on environmental conditions and may help guide future fish-feed strategies to support the health and resilience of farmed Atlantic salmon.

## 1. Introduction

Fish oil is the best source of fatty acids in aquafeed formulation. It contains high levels of n-3 long-chain polyunsaturated fatty acids. These fatty acids are essential for fish growth and health. Two important ones are EPA (eicosapentaenoic acid) and DHA (docosahexaenoic acid). However, the availability of fish oil is constrained by the finite supply of marine raw materials, and continued reliance on marine-derived ingredients raises sustainability concerns associated with the exploitation of wild fish resources. The aquaculture industry has tried to use vegetable oils instead. Vegetable oils are cheaper and more sustainable, but they lack EPA and DHA [1]. Atlantic salmon cannot convert vegetable oils into enough EPA and DHA [2]. Therefore, EPA and DHA must be added to the diet. The dietary requirements for EPA and DHA are not fixed. It depends on the fish’s growth stage and environment [3]. Relatively low dietary levels may be sufficient under normal conditions, whereas higher levels may be required under stressful conditions [4].

Farming Atlantic salmon involves multiple aquaculture-related stressors, including crowding, handling, and adverse environmental conditions [5]. These stressors are often unpredictable and may occur at irregular intervals. Such exposure can be modeled experimentally as unpredictable chronic stress (UCS). UCS is a good model for real farming conditions [6]. In UCS protocols, fish are exposed to different stressors in an unpredictable sequence to minimize habituation. This approach is intended to induce a sustained stress response. Previous studies have applied UCS protocols to Atlantic salmon. Løvmo et al. investigated the effects of different dietary EPA and DHA levels in Atlantic salmon subjected to UCS [2,7]. These studies evaluated intestinal health and demonstrated diet-dependent responses to UCS. A diet low in highly unsaturated fatty acids (HUFAs; 8.5 g/kg of EPA+DHA) was associated with impaired intestinal health following UCS, including increased inflammation and compromised intestinal barrier function. The fish had inflammation and poor gut barrier function [7]. In contrast, high-HUFA diets were associated with improved stress resilience. Both a high-DHA diet and a high-EPA diet improved stress resistance [2]. This suggests that EPA and DHA can help fish cope with stress. However, whether and how EPA and DHA exert differential effects remain unclear. Previous studies have primarily evaluated growth performance under standard or non-stressful rearing conditions [8]. These studies generally did not examine stress responses or directly compare the effects of EPA and DHA. Consequently, the physiological importance of EPA and DHA under aquaculture-related stress conditions may not be fully reflected by studies conducted exclusively under non-stressful conditions.

The liver is a key organ for metabolism. It responds strongly to diet and to stress. Therefore, the liver represents an appropriate target organ for investigating how EPA and DHA exert their effects. Characterizing these responses at the transcriptional level may therefore provide mechanistic insight. Some studies have looked at this under normal conditions. For example, Xue and colleagues studied the liver of Atlantic salmon [9]. They demonstrated that dietary DHA and EPA levels differentially affected hepatic gene expression. But they did not use stress. Therefore, the hepatic responses to dietary EPA and DHA under UCS remain unclear. For example, previous UCS studies in Atlantic salmon have applied a range of stressors, including chasing, netting, temperature shocks, noise and light disturbance, brief hypoxia, and tank emptying, in an unpredictable sequence over a 3-week period [6]. Similarly, a previous study in largemouth bass (*Micropterus salmoides*) examined the effects of dietary DHA on antioxidant responses [10]. They found that DHA was better for basal antioxidant enzymes. But again, that study did not include an unpredictable chronic stress challenge. So their results may not apply to farming conditions. Stress changes the way the body uses fats [11]. It also changes antioxidant defenses [12]. The body may need to produce more energy [13]. It may need to fight oxidative damage [12]. These long-chain n-3 fatty acids may help with these processes. However, whether EPA and DHA exert distinct hepatic regulatory effects during chronic stress remains unresolved. There is also a possible trade-off. High amounts of EPA or DHA may boost metabolism. But they might also reduce cell protection. For example, they could lower the production of heat shock proteins like HSP90. HSP90 helps cells survive stress [14]. However, it is unknown whether high-HUFA intake affects HSP90. This hypothesis was investigated in the present study.

Specifically, the present study aims to systematically compare the hepatic transcriptomic and gene expression responses of Atlantic salmon fed high-DHA or high-EPA diets under both unstressed and UCS conditions, and to elucidate the distinct molecular mechanisms by which dietary EPA and DHA differentially modulate lipid metabolism, antioxidant defense, and stress adaptation in the liver. By improving our understanding of the effects of dietary EPA and DHA under different farming conditions, we hope to contribute to the development of better fish feeds for Atlantic salmon.

## 2. Materials and Methods

### 2.1. Fish and Experimental Design

The experimental procedures were approved by the Norwegian Experimental Animal Committee (FOTS ID: 15041). In brief, Atlantic salmon post-smolts with an average body weight of 97.4 g were transferred from Lerøy Lensvik to the NTNU (Norwegian University of Science and Technology) Sealab facility (Trondheim, Norway) and acclimated for over 6 weeks in flow-through seawater under a 24 h light cycle and fed a commercial diet manufactured by BioMar. The experimental diets were manufactured at NTNU Sealab from uncoated feed produced by BioMar and were vacuum-coated with specific oil blends. After acclimatization, fish were given two trial periods: a 10-week feeding period and a 3-week unpredictable chronic stress (UCS) period. When the trial started, fish were randomly divided into 3 groups in 400 L tanks with flow-through seawater of about 8.2 °C. Each tank contained 25 randomly allocated fish. Each dietary treatment was represented by three randomly positioned replicate tanks. Fish were fed to apparent satiation twice daily for 2 h, with at least 10% excess feed remaining in the tank outlet after feeding. Fish were fed diets differing in HUFA composition. The ingredient formulation, oil-blend composition, crude lipid content, and selected analyzed fatty acid composition of the experimental diets are presented in Table 1. The crude lipid contents were 20.2%, 19.5%, and 19.0% in the LF, DF, and EF diets, respectively. The corresponding EPA+DHA contents were 8.5, 35.2, and 33.1 g/kg of the diet, respectively. The final HUFA compositions of the diets were as follows: low-HUFA (LF) diet with EPA+DHA at 8.5 g/kg of the diet and 4.53% of total FA, high-DHA diet (DF) with 28.7 g/kg and 15.86% of total FA, and high-EPA (EF) diet with 25 g/kg and 14.17% of total FA [2]. The diet formulation and composition data for these experimental diets were previously reported by Løvmo et al. (2021) [2]. During the 3-week UCS period, three stressors were randomly selected each day from a set of six stressors: crowding, chasing with a net, light disturbance, sound disturbance, chasing with a cane, and hypoxia. The type and timing of the stressors were randomized daily to maximize unpredictability. During the 3-week UCS period, fish continued to receive their respective experimental diets (LF, DF, and EF) [2]. After the 3-week UCS period, the low-HUFA, DHA-rich, and EPA-rich diet groups were designated as LU, DU, and EU, respectively.

### 2.2. Sampling

Fish were sampled at two timepoints: the first sampling happened after the feeding period, while the second sampling occurred after the UCS period. At each sampling timepoint, five fish from each experimental tank were sampled in each of the two sampling rounds, and body weight and length were recorded. The fish were fasted for 24 h before sampling and four fish from each group were randomly selected and euthanized with anesthetic Finquel.vet^®^ (100 mg L^−1^ Tricaine mesylate, Scanvacc, Hvam, Norway), after which liver samples were collected. Liver samples were placed in 2 mL Eppendorf tubes containing RNAlater™ Stabilization Solution (Invitrogen, Waltham, MA, USA, Cat. No. AM7020), stored at 4 °C for 24 h before storage at −80 °C.

### 2.3. Growth Calculations

Weight gain (WG, %) was calculated as [(*W*_2_ − *W*_1_)/*W*_1_] × 100, and specific growth rate (SGR, % day^−1^) was calculated as [ln(*W*_2_) − ln(*W*_1_)] × 100/*t*, where *W*_1_ and *W*_2_ are the initial and final body weights (g), respectively, and *t* is the number of experimental days. Differences in growth performance among dietary treatments were analyzed using one-way ANOVA followed by Tukey’s honestly significant difference (HSD) post hoc test, with *p* < 0.05 considered statistically significant.

$$\left[\mathrm{WG}\;\left(\%\right)=\frac{W_{2}-W_{1}}{W_{1}}\times 100\right]$$

$$\left[\mathrm{SGR}\;\left(\%\;{\mathrm{day}}^{-1}\right)=\frac{\ln W_{2}-\ln W_{1}}{t}\times 100\right]$$

### 2.4. RNA Extraction and Quantification

RNA sequencing was performed on 24 liver samples representing LF, DF, EF, and their corresponding UCS-exposed groups (LU, DU, and EU; *n* = 4 per group). Liver samples were placed on ice and 1 mL of an RLT Lysis buffer (RNeasy Mini kit) mixed with b-mercaptoethanol (1% in RLT) was added before homogenization using a rotor–stator homogenizer (Polytron Kinematica PT3000, Kinematica AG, Malters, Switzerland) for up to 10 s at maximum speed. After homogenization, RNeasy Mini kit (QIAGEN GmbH, Hilden, Germany, Cat. No. 74104) and RNase-Free DNase set (Qiagen, Cat. No. 79256) were used for RNA isolation and DNase treatment as described in the manual. The integrity and purity of total RNA were evaluated with a Bioanalyzer 2100 instrument and RNA 6000 Nano Assay Kit (both from Agilent Technologies, Inc., Santa Clara, CA, USA). Samples with RNA integrity number (RIN) ≥ 7 were accepted. RNA concentration was measured with Nanodrop 2000c Spectrophotometer (Thermo Scientific, Waltham, MA, USA).

### 2.5. Transcriptome Analysis

RNA sequencing (*n* = 4 per group) was performed by BGI Genomics (Hong Kong). The raw data of transcriptome was submitted to NCBI Sequence Read Archive (SRA) under accession No. PRJNA1458987. Reads were filtered for quality and mapped to the *Salmo salar* reference genome (ICSASG_v2) using HISAT2. Gene expression was quantified with RSEM, and differentially expressed genes (DEGs) were identified using DESeq2 (|log2FC| ≥ 1, adj. *p* ≤ 0.05). Functional enrichment was performed using GO via clusterProfiler. Reads were filtered for quality and mapped to the *Salmo salar* reference genome (Ssal_v3.1) using STAR 2.7.11 and KEGG was performed for functional enrichment.

### 2.6. qRT-PCR and Statistical Analysis

The genes examined by qRT-PCR included eukaryotic translation elongation factor 1 alpha (*EF1α*), eukaryotic translation elongation factor 1 alpha B (*EF1αB*), beta-actin (*β-actin*), apolipoprotein A-I (*APOA1*), apolipoprotein A-IV (*APOA4*), fatty acid transport protein (*FATP*), peroxisome proliferator-activated receptor alpha (*PPARα*), peroxisome proliferator-activated receptor gamma (*PPARγ*), heat shock protein 70 (HSP70), heat shock protein 90 beta (*HSP90b*), tumor necrosis factor alpha (*TNFα*), transforming growth factor beta 1/5 (*TGFβ1/5*), catalase (*CAT*), superoxide dismutase (*SOD*), glutathione peroxidase (*GPX*), glutathione peroxidase 4b (*GPX4b*), glutathione peroxidase 7 (*GPX7*), glutathione reductase (*GR*), sterol regulatory element-binding protein 1 (*SREBP1*), sterol regulatory element-binding protein 2 (*SREBP2*), 3-hydroxy-3-methylglutaryl-CoA reductase (*HMGCR*), and fatty acid synthase (*FASN*).

Quantitative RT-PCR (qRT-PCR) was carried out on a Roche LightCycler 480 instrument (Roche Diagnostics Ltd., Rotkreuz, Switzerland). *EF1α*, *EF1αB*, and *β-actin* were evaluated as candidate reference genes, and β-actin was selected as the reference gene for normalization according to previous publication [15]. Each 15 μL reaction mixture consisted of 7.5 μL of BioEasy SYBR Green master mix (Bio-Rad), 3.1 μL of nuclease-free water, 4 μL of diluted cDNA (200 ng), and 0.2 μL of each gene-specific primer (10 μM) (Table 2). Statistical analysis was performed using an unpaired, two-tailed Student’s *t*-test. Differences were considered significant at *p* ≤ 0.05.

## 3. Results

### 3.1. Growth Performance

At the start of the trial, mean body weights ranged from 149 g to 151 g among the three dietary groups. After the 10-week feeding period, mean body weights reached 388 g, 392 g, and 392 g in the LF, DF, and EF groups, respectively, corresponding to weight gains of 157%, 163%, and 163% and specific growth rates of 1.36, % 1.41%, and 1.37% day^−1^, respectively. No significant differences in growth performance were detected among dietary groups. Following the 3-week UCS period, mean body weights were 396 g in LU, 402 g in DU, and 410 g in EU, with corresponding weight gains of 2.0%, 4.3%, and 4.6%. Growth was markedly attenuated during UCS, but no significant differences were observed among dietary groups.

### 3.2. RNA Sequencing and Transcriptome Analysis

RNA sequencing was performed on 24 liver samples from the six experimental groups. When summarized by group, the mean numbers of clean reads ranged from 43.24 to 44.02 million, and the mean total clean bases ranged from 4.32 to 4.40 Gb. Mean Q20 and Q30 values ranged from 98.17% to 98.61% and from 94.49% to 95.63%, respectively, while the mean clean-read ratio ranged from 92.63% to 93.57%. The mean rRNA ratio ranged from 0.61% to 2.46% (Table 3), indicating that the sequencing data were of sufficient quality for downstream analysis. After alignment to the Atlantic salmon reference genome, the mean total mapping ratio ranged from 93.99% to 95.05%, while the mean unique mapping ratio ranged from 67.55% to 68.65% among groups (Table 4). Mapping efficiency was generally consistent among groups.

At the group level, the mean number of detected genes ranged from 32,039 to 35,842, including 29,180–32,438 known genes and 2859–3405 novel genes. The mean total number of transcripts ranged from 46,735 to 56,288, comprising 29,401–35,631 known transcripts and 17,334–20,709 novel transcripts (Table 5).

### 3.3. Effects of Dietary HUFAs Under Unstressed Conditions

#### 3.3.1. Differentially Expressed Genes Under Unstressed Conditions

**Figure 1:**
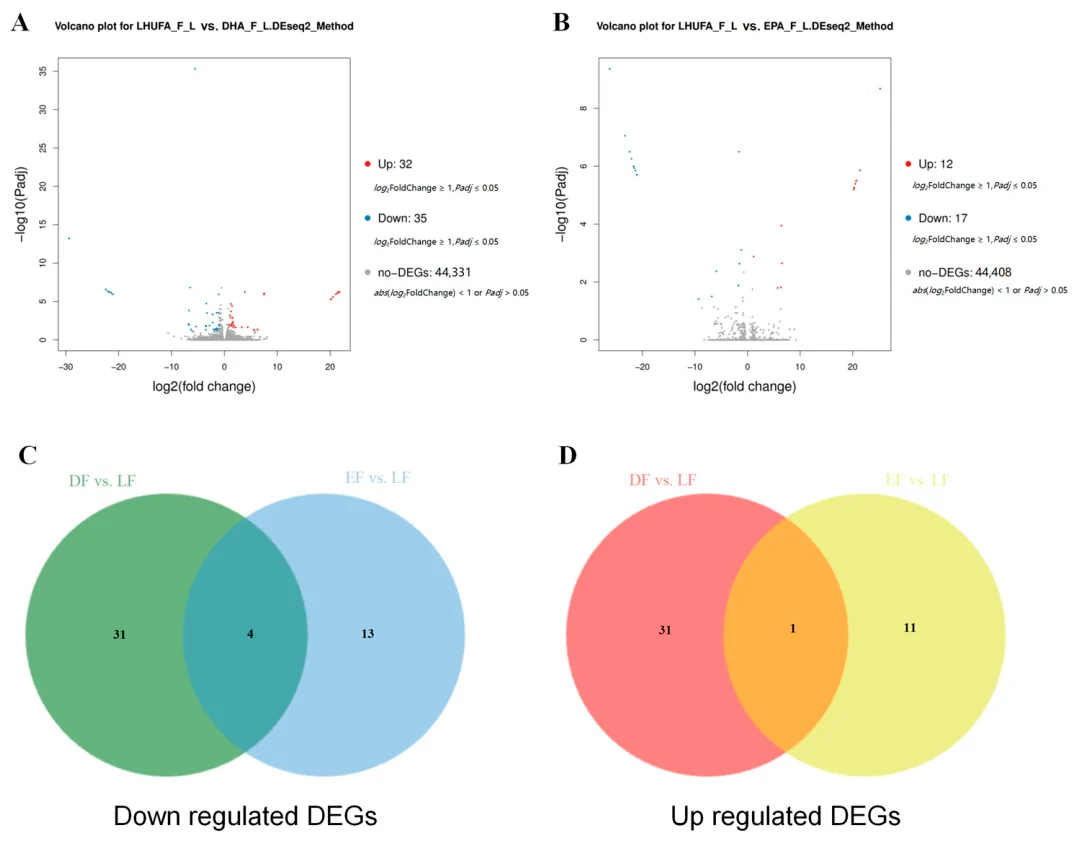
Hepatic differentially expressed genes in Atlantic salmon under unstressed conditions. Volcano plots for DF versus LF (**A**) and EF versus LF (**B**). Venn diagrams show the overlap of downregulated (**C**) and upregulated (**D**) DEGs between the two comparisons. DEGs were identified using |log2 fold change| ≥ 1 and adjusted *p* ≤ 0.05.

Relative to LF, DF resulted in 67 hepatic DEGs, including 32 upregulated and 35 downregulated genes, whereas EF resulted in 29 DEGs, including 12 upregulated and 17 downregulated genes (Figure 1A,B). The two comparisons shared four downregulated and one upregulated DEG. DF had 31 unique downregulated and 31 unique upregulated DEGs, whereas EF had 13 unique downregulated and 11 unique upregulated DEGs (Figure 1C,D). Thus, under unstressed conditions, the DF vs. LF and EF vs. LF comparisons differed in the number and composition of DEGs. Among the significant DEGs, several transcripts showed particularly large absolute fold changes. In DF vs. LF, prominent DEGs included albumin 2 precursor, ER membrane protein complex subunit 10-like, and hemoglobin subunit alpha, whereas in EF vs. LF, cytochrome c oxidase subunit I, 60S ribosomal protein L23a (rl23a), and ribosomal protein L23a were among the most prominent. These genes are mainly associated with lipid binding, membrane function, oxygen transport, mitochondrial respiration, and translation.

#### 3.3.2. GO Functional Classification Under Unstressed Conditions

**Figure 2:**
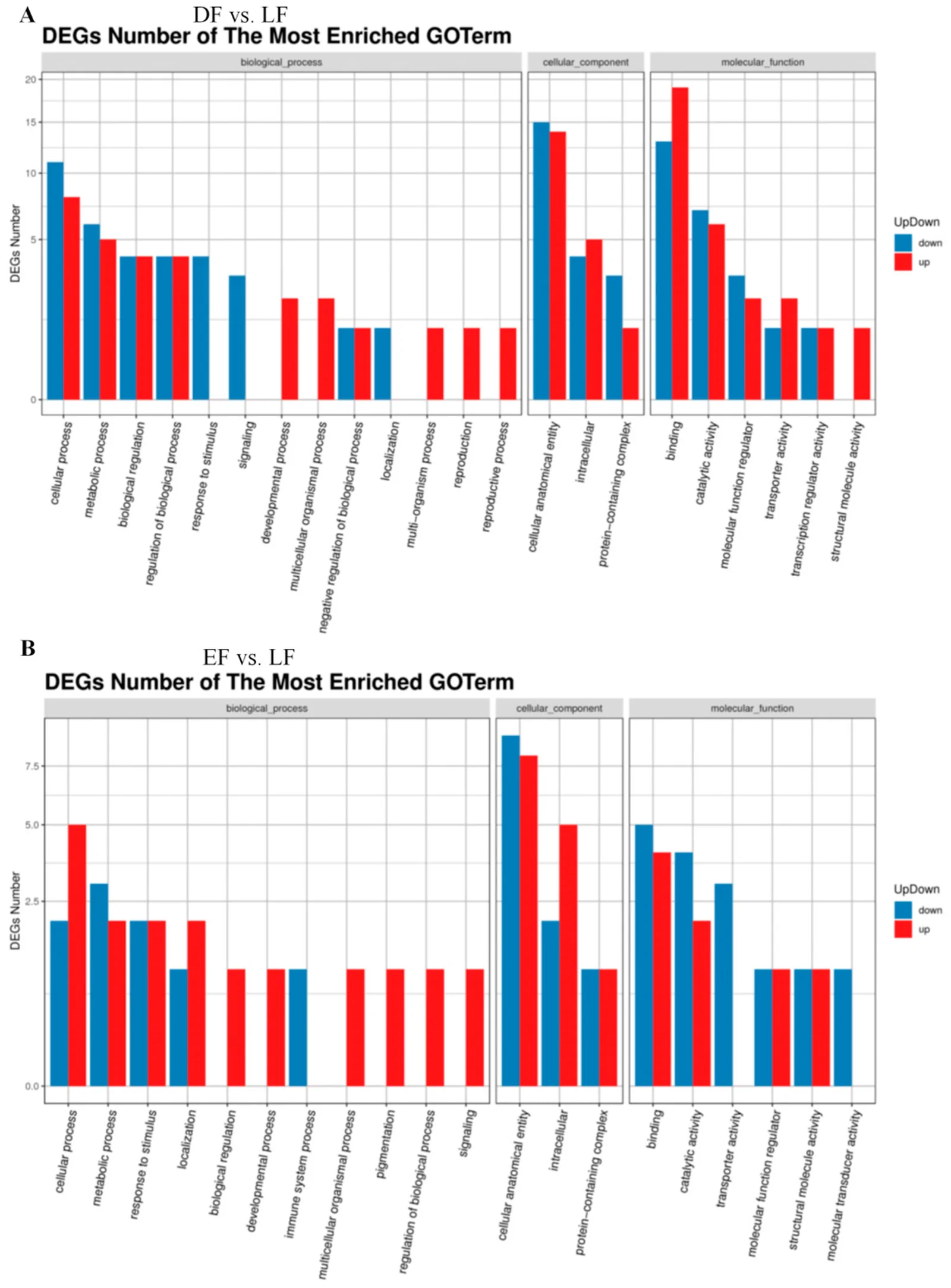
GO functional classification of hepatic DEGs in Atlantic salmon under unstressed conditions. DEGs are grouped into biological process, cellular component, and molecular function categories for DF versus LF (**A**) and EF versus LF (**B**). Red and blue bars indicate upregulated and downregulated DEGs, respectively. GO categories were arranged in the order of BP, CC, and MF, with categories within each ontology domain ranked according to their enrichment levels.

In the DF vs. LF comparison, DEGs assigned to the biological process domain were mainly classified into cellular process, metabolic process, biological regulation, and regulation of biological process. Within the cellular component domain, most DEGs were assigned to cellular anatomical entity and intracellular, whereas binding and catalytic activity were the predominant molecular function categories (Figure 2A).

The EF vs. LF comparison showed a broadly similar GO distribution but contained fewer DEGs. Cellular process, metabolic process, response to stimulus and localization were the largest biological process categories; cellular anatomical entity and intracellular predominated in the cellular component domain; and binding and catalytic activity were the principal molecular function categories (Figure 2B).

#### 3.3.3. KEGG Pathway Enrichment Under Unstressed Conditions

**Figure 3:**
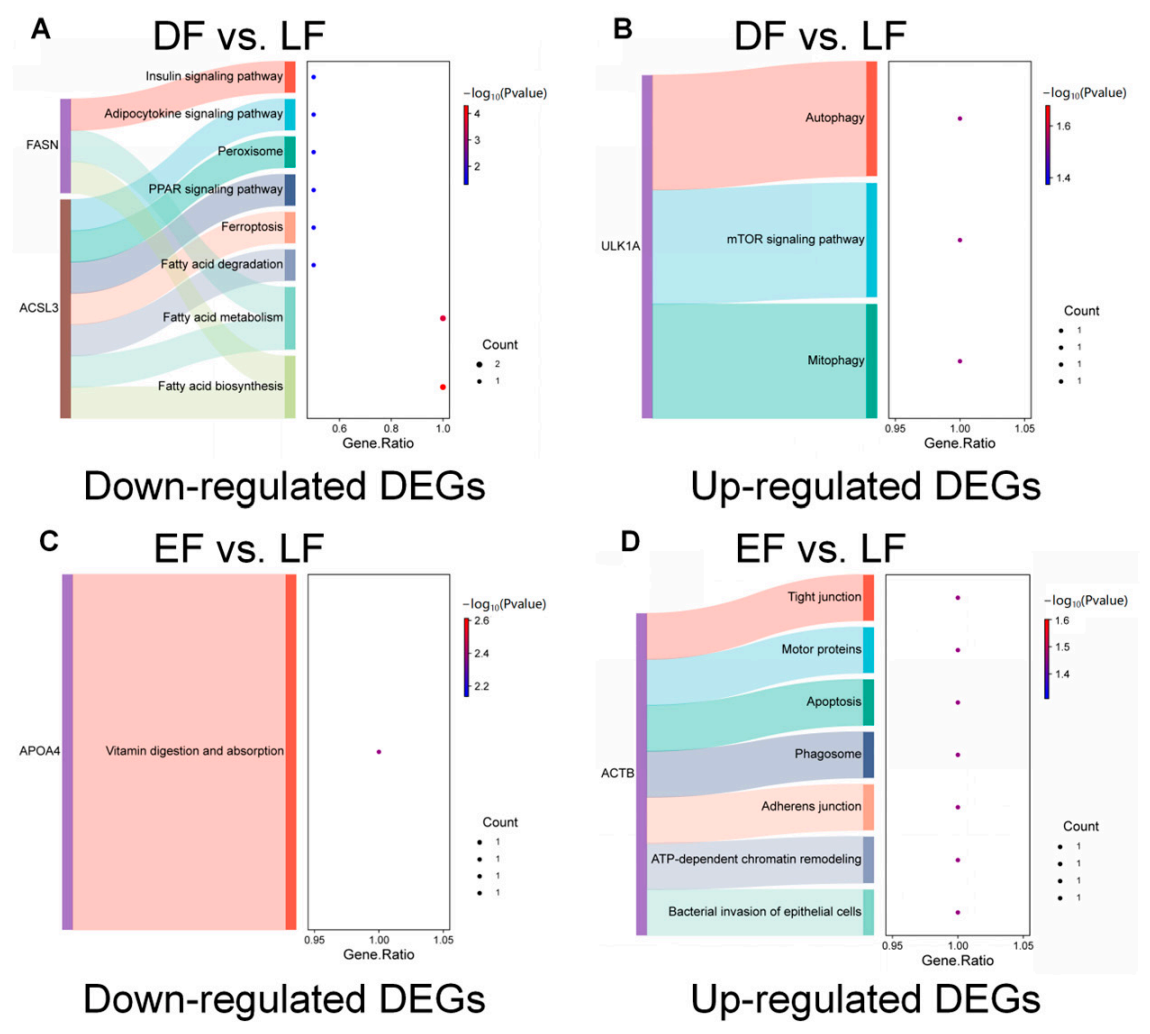
KEGG pathway enrichment of hepatic DEGs in Atlantic salmon under unstressed conditions. Downregulated (**A**) DEGs in DF versus LF, and upregulated (**B**) DEGs in DF versus LF. Downregulated (**C**) DEGs in EF versus LF, and upregulated (**D**) DEGs in EF versus LF. Sankey links connect DEGs with enriched pathways; point size represents gene count and color represents −log10(*p* value).

KEGG pathway enrichment analysis was conducted, and Sankey plots were drawn to illustrate the associations between DEGs and their annotated pathways, highlighting the involvement of key genes in multiple pathways. As shown in Figure 3 in the DF vs. LF comparison, the downregulated DEG *FASN* was associated with the insulin signaling pathway, fatty acid metabolism, and fatty acid biosynthesis, while *ACSL3* was associated with adipocytokine signaling pathway, peroxisome, PPAR signaling pathway, ferroptosis, fatty acid degradation, fatty acid metabolism, and fatty acid biosynthesis (Figure 3A). The upregulated gene *ULK1A* was associated with autophagy, the mTOR signaling pathway, and mitophagy (Figure 3B).

In the EF vs. LF comparison, downregulated *APOA4* was associated with vitamin digestion and absorption (Figure 3C). Upregulated *ACTB* was linked to tight junction, motor proteins, apoptosis, phagosome, adherens junction, ATP-dependent chromatin remodeling, and bacterial invasion of epithelial cells (Figure 3D).

#### 3.3.4. qRT-PCR Validation Under Unstressed Conditions

**Figure 4:**
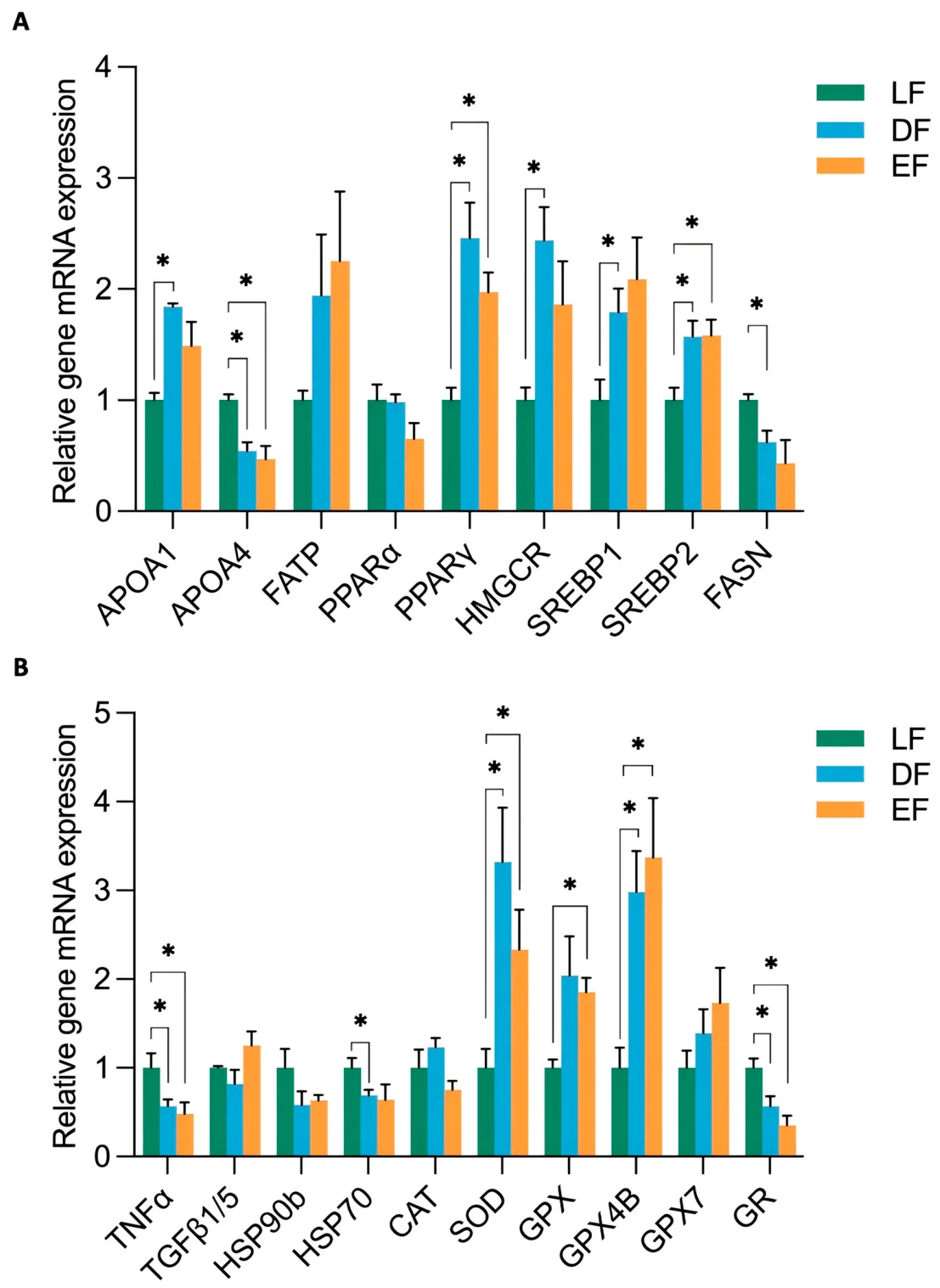
Relative mRNA expression of hepatic genes in Atlantic salmon under unstressed conditions. Lipid metabolism-related genes (**A**) and oxidative stress- and immune-related genes (**B**) were measured by qRT-PCR. Expression was normalized to β-actin and is presented relative to LF, which was set to 1. Values are means ± SD (*n* = 4). *, *p* < 0.05 versus LF.

Representative genes involved in hepatic lipid transport and metabolic regulation, cholesterol biosynthesis and homeostasis (Figure 4A), inflammation, and antioxidant defense against oxidative and lipid peroxidative stress (Figure 4B) were analyzed by qRT-PCR to validate the RNA-seq results and further evaluate the effects of dietary HUFAs on the salmon liver. The qRT-PCR results showed distinct diet-dependent transcriptional responses (Figure 4). Relative to LF, DF significantly increased the expression of APOA1, PPARγ, HMGCR, SREBP1 and SREBP2, and significantly decreased expression of APOA4 and FASN, whereas, EF significantly increased PPARγ and SREBP2, and significantly decreased APOA4 expression (Figure 4A). Among the oxidative stress- and immune-related genes, DF significantly increased *SOD* and *GPX4b*, and significantly decreased *TNFα*, *HSP70* and *GR* expression, whereas EF significantly increased *SOD*, *GPX* and *GPX4b*, and significantly decreased *TNFα* and *GR* expression (Figure 4B). None of the other genes examined differed significantly from LF.

### 3.4. Effects of Dietary HUFAs Under Unpredictable Chronic Stress

#### 3.4.1. Differentially Expressed Genes Under UCS

**Figure 5:**
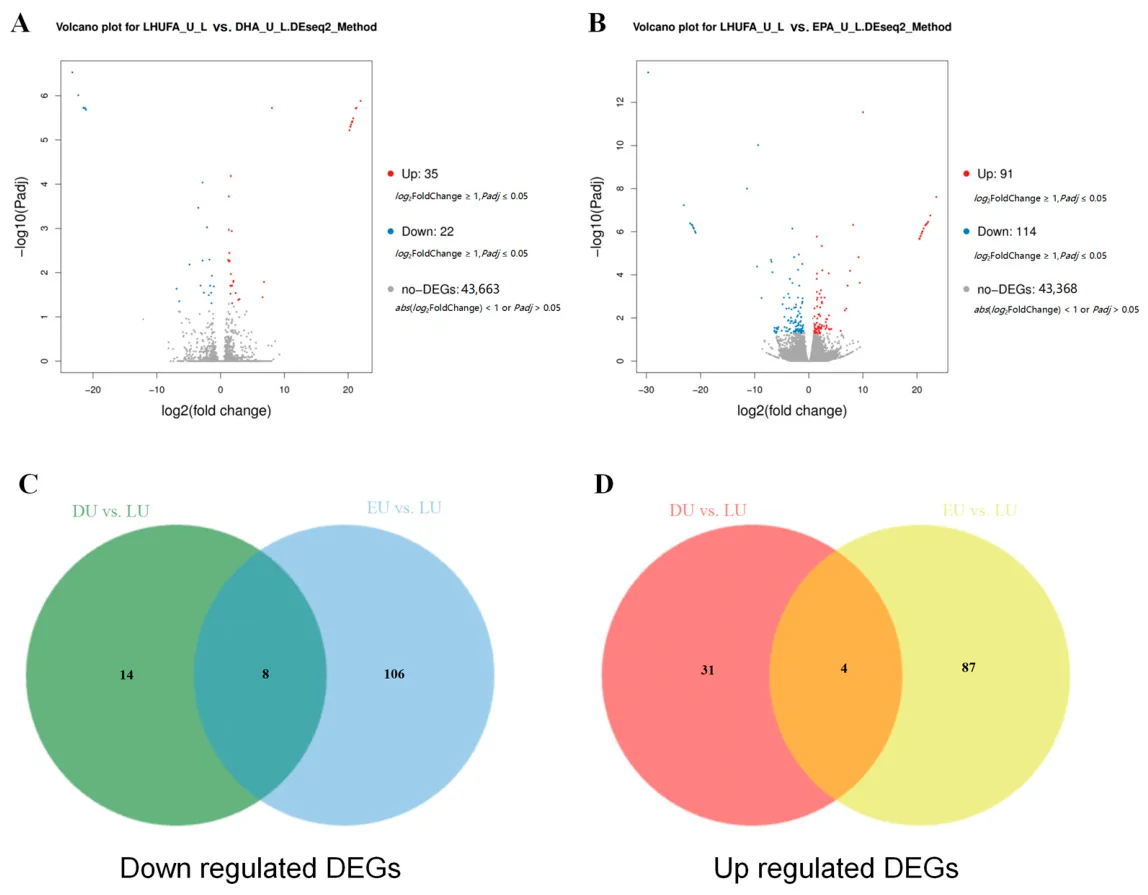
Hepatic differentially expressed genes in Atlantic salmon after UCS. Volcano plots for DU versus LU (**A**) and EU versus LU (**B**). Venn diagrams show the overlap of downregulated (**C**) and upregulated (**D**) DEGs between the two comparisons. DEGs were identified using |log2 fold change| ≥ 1 and adjusted *p* ≤ 0.05.

Following UCS, the DU vs. LU comparison contained 57 hepatic DEGs, including 35 upregulated and 22 downregulated genes, whereas the EU vs. LU comparison contained 205 DEGs, including 91 upregulated and 114 downregulated genes (Figure 5A,B). The two comparisons shared eight downregulated and four upregulated DEGs. DU had 14 unique downregulated and 31 unique upregulated DEGs, whereas EU had 106 unique downregulated and 87 unique upregulated DEGs (Figure 5C,D). Thus, the DU vs. LU and EU vs. LU comparisons differed markedly in the number and composition of the detected DEGs. Several significant DEGs also showed particularly large absolute fold changes after UCS. In DU vs. LU, prominent examples included rl23a, alpha-2-macroglobulin-like, and proteinase-activated receptor 1-like, while in EU vs. LU, albumin 2 precursor, rl23a, and proteinase-activated receptor 1-like were among the most prominent. These genes are related to translation, protease inhibition, coagulation-related signaling, and lipid binding.

#### 3.4.2. GO Functional Classification Under UCS

**Figure 6:**
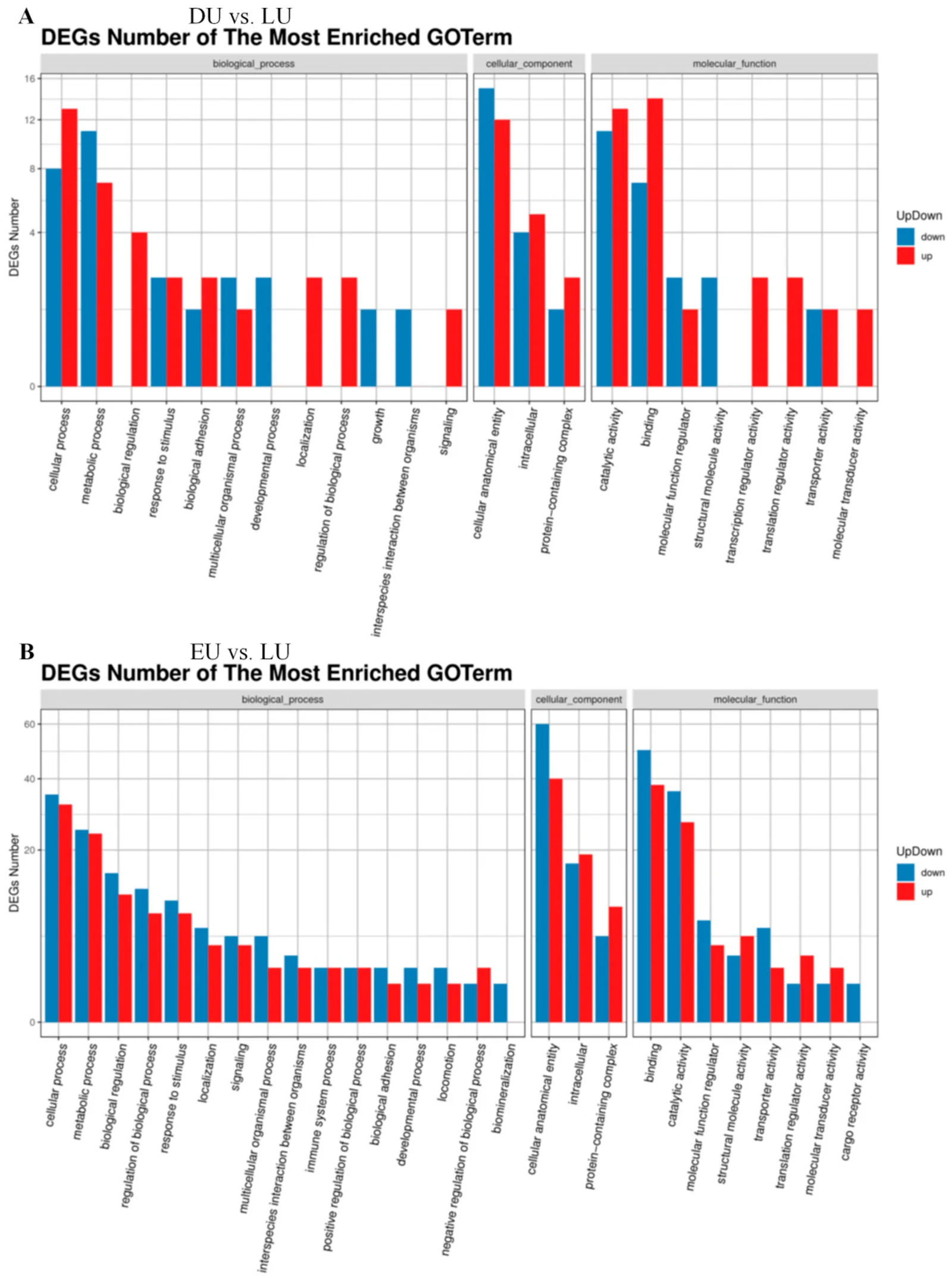
GO functional classification of hepatic DEGs in Atlantic salmon after UCS. DEGs are grouped into biological process, cellular component, and molecular function categories for DU versus LU (**A**) and EU versus LU (**B**). Red and blue bars indicate upregulated and downregulated DEGs, respectively. GO categories were arranged in the order of BP, CC, and MF, with categories within each ontology domain ranked according to their enrichment levels.

In the DU vs. LU comparison, cellular process and metabolic process contained the largest numbers of DEGs in the biological process domain. Cellular anatomical entity and intracellular predominated within the cellular component domain, while binding and catalytic activity were the major molecular function categories (Figure 6A).

The EU vs. LU comparison contained substantially more DEGs across GO categories. The principal biological process categories included cellular process, metabolic process, biological regulation, regulation of biological process and response to stimulus. Cellular anatomical entity and intracellular were the dominant cellular component categories, and binding and catalytic activity accounted for the largest molecular function categories (Figure 6B).

#### 3.4.3. KEGG Pathway Enrichment Under UCS

**Figure 7:**
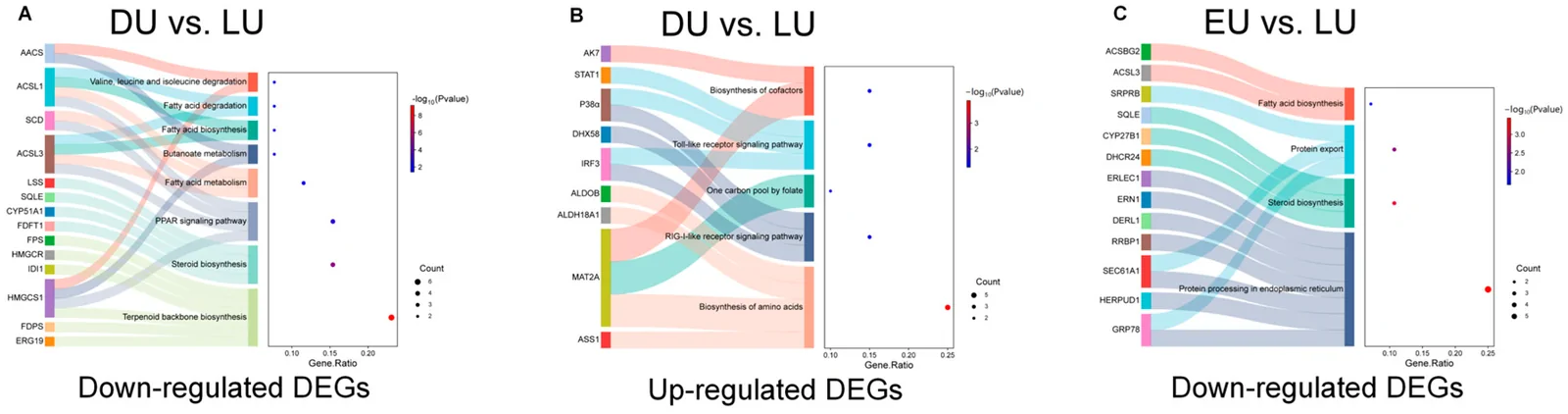
KEGG pathway enrichment of hepatic DEGs in Atlantic salmon after UCS. Downregulated (**A**) and upregulated (**B**) DEGs in DU versus LU, and downregulated (**C**) DEGs in EU versus LU. Sankey links connect DEGs with enriched pathways; point size represents gene count and color represents −log10(*p* value).

In the DU vs. LU comparison, downregulated DEGs were associated with valine, leucine and isoleucine degradation, fatty acid degradation, fatty acid biosynthesis, butanoate metabolism, fatty acid metabolism, the PPAR signaling pathway, steroid biosynthesis, and terpenoid backbone biosynthesis (Figure 7A). Upregulated DEGs were associated with biosynthesis of cofactors, the Toll-like receptor signaling pathway, one-carbon pool by folate, the RIG-I-like receptor signaling pathway, and biosynthesis of amino acids (Figure 7B).

In the EU vs. LU comparison, downregulated DEGs were associated with fatty acid biosynthesis, protein export, steroid biosynthesis, and protein processing in the endoplasmic reticulum (Figure 7C). No KEGG pathways were significantly enriched among the 91 upregulated DEGs in the EU vs. LU comparison. This does not indicate that these genes lacked KEGG annotations, but rather that no pathway reached the significance threshold in the enrichment analysis. This may occur when the DEGs are distributed across multiple functional pathways rather than being concentrated within a limited number of KEGG pathways.

#### 3.4.4. qRT-PCR Validation Under UCS

**Figure 8:**
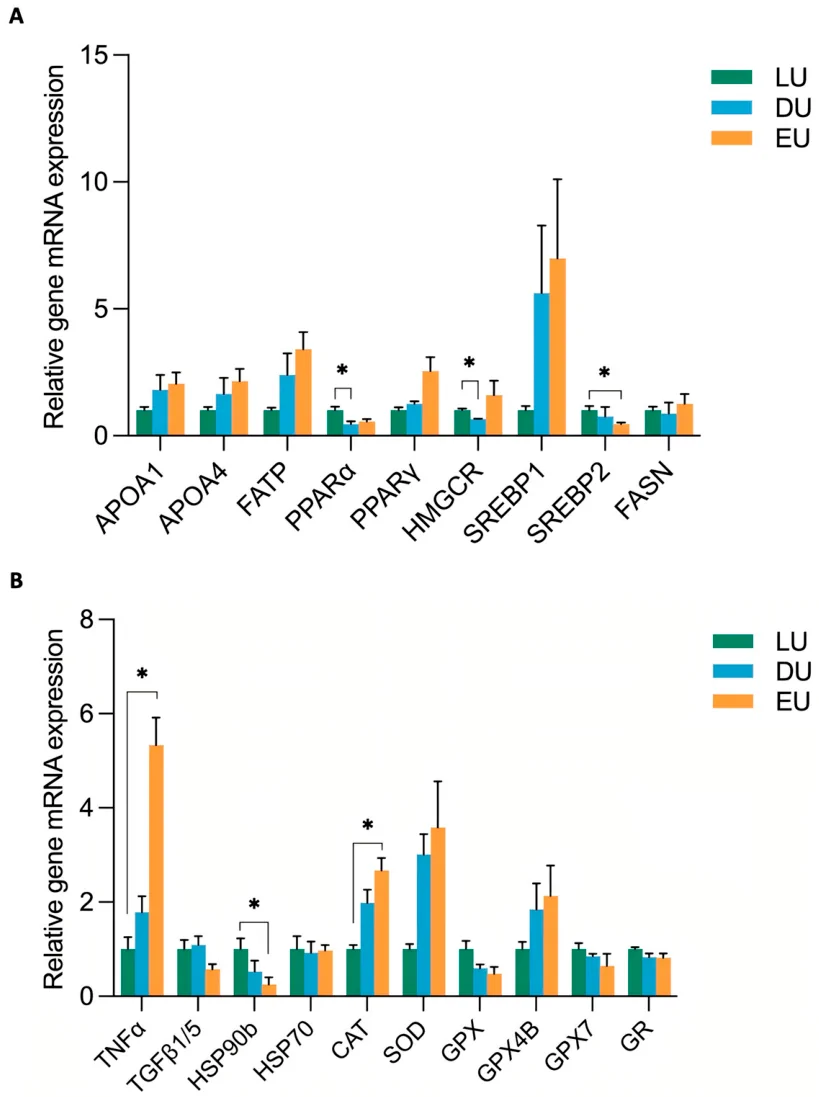
Relative mRNA expression of hepatic genes in Atlantic salmon after UCS. Lipid metabolism-related genes (**A**) and oxidative stress-, immune-, and apoptosis-related genes (**B**) were measured by qRT-PCR. Expression was normalized to β-actin and is presented relative to LU, which was set to 1. Values are means ± SD (*n* = 4). *, *p* < 0.05 versus LU.

Under UCS, compared with LU, DU significantly decreased expression levels of *PPARα* and *HMGCR*; meanwhile EU significantly decreased expression levels of *SREBP2* (Figure 8A). EU significantly increased *TNFα* and *CAT*, and significantly decreased HSP90b expression (Figure 8B).

## 4. Discussion

In the present study, the impacts of DHA-rich and EPA-rich diets on the liver transcriptome of Atlantic salmon were evaluated under both unstressed and UCS conditions. Atlantic salmon post-smolts were fed a low-EPA+DHA diet (LF group), a DHA-rich diet (DF group), or an EPA-rich diet (EF group) for 10 weeks, followed by 3 weeks of UCS (LU, DU, EU groups). Liver transcriptomes were analyzed after the feeding and UCS periods, and the expression of selected genes was measured by qRT-PCR. The analysis focused on pathways and genes involved in fatty acid metabolism, autophagy, hepatic lipid transport and metabolic regulation, cholesterol biosynthesis and homeostasis, steroid biosynthesis, and antioxidant defense against oxidative stress and lipid peroxidation. These findings reveal distinct hepatic regulatory responses to DHA- and EPA-enriched diets in Atlantic salmon liver under UCS and provide a basis for future studies aimed at optimizing dietary EPA and DHA provision across production conditions.

### 4.1. Context-Dependent Hepatic Transcriptional Responses to Dietary DHA and EPA in the Absence of Significant Growth Differences

The present study shows that dietary DHA and EPA were associated with distinct hepatic transcriptional responses and that the relative extent of these responses depended on the environmental context. Under unstressed conditions, DF produced more DEGs than EF (67 vs. 29), whereas after UCS the pattern was reversed, with more DEGs in EU than in DU (205 vs. 57). Only five DEGs were shared between the two high-HUFA diets under unstressed conditions and 12 after UCS, indicating that the responses to DHA- and EPA-rich diets were largely non-overlapping. These molecular differences occurred without significant differences in growth among dietary groups [2]. Thus, within the duration and statistical power of the trial in the present study, transcriptomic sensitivity was greater than growth sensitivity. A similar dissociation between conventional performance traits and hepatic molecular responses has been observed in transcriptomic studies of Atlantic salmon exposed to graded dietary DHA levels [16].

The switch in the number of DEGs from a broader DHA-associated response before stress to a broader EPA-associated response after stress suggests context-dependent nutrient regulation rather than a fixed ranking of DHA and EPA. This interpretation is consistent with previous Atlantic salmon studies showing that dietary EPA+DHA can alter hepatic lipid-metabolic, redox, and immune transcripts even when conventional performance indicators show limited separation among adequate dietary treatments [9,17,18]. However, the absolute number of detected DEGs should be interpreted cautiously, as it may be influenced by biological variability, sample preparation and processing, sequencing characteristics, statistical power, and the thresholds used for differential-expression analysis. Therefore, DEG counts alone should not be interpreted as a direct measure of the magnitude or biological importance of the response. Therefore, pathway-level analyses and qRT-PCR results are necessary to interpret the biological significance of these differences.

### 4.2. DHA- and EPA-Associated Responses Under Unstressed Conditions

Under unstressed conditions, the DHA-rich diet elicited the broader hepatic response. In DF vs. LF, *FASN* and *ACSL3* were downregulated and linked to fatty acid synthesis/metabolism pathways, while *ULK1A* was upregulated and associated with autophagy, mTOR signaling, and mitophagy. Together, these observations are compatible with feedback suppression of endogenous lipid synthesis when preformed long-chain n-3 PUFA is abundant, consistent with the nutritional regulation of SREBP and fatty acid biosynthetic genes reported in Atlantic salmon cells [19,20]. Dietary fatty acid composition is known to modify hepatic fatty acid uptake, oxidation, and metabolic partitioning in Atlantic salmon [21]. Dietary regulation of lipid-biosynthetic genes in salmonids is well established, and hepatic transcription can respond to changes in dietary lipid and long-chain n-3 PUFA supply independent of growth [9,22,23,24].

The qRT-PCR data further distinguished the basal responses. DF increased APOA1, *PPARγ*, *HMGCR*, *SREBP1*, and *SREBP2* and decreased *APOA4* and *FASN*, whereas EF increased *PPARγ* and *SREBP2* and decreased *APOA4*. The simultaneous induction of sterol-regulatory transcripts and reduction in *FASN* in DF suggest selective remodeling of lipid transport, cholesterol homeostasis, and de novo fatty acid synthesis rather than a uniform increase or decrease in hepatic lipogenesis [22,25,26]. Both diets also modified redox-related genes: DF increased *SOD* and *GPX4b*, whereas EF increased *SOD*, *GPX*, and *GPX4b*; both reduced *TNFα* and *GR*. These changes indicate that adequate DHA or EPA supply could alter mRNA expression related to basal antioxidant and immune tone [10,27].

### 4.3. Diet-Associated Hepatic Responses During UCS

After UCS, DU showed coordinated suppression of several metabolic processes relative to LU. Downregulated DEGs were associated with fatty acid degradation and biosynthesis, branched-chain amino-acid degradation, butanoate metabolism, PPAR signaling, steroid biosynthesis, and terpenoid backbone biosynthesis. Consistent with these enrichments, qRT-PCR showed lower *PPARα* and *HMGCR* expression in DU. This pattern suggests reduced transcriptional investment in both lipid catabolism and sterol synthesis rather than a simple stimulation of energy mobilization. Conversely, enrichment of Toll-like receptor and RIG-I-like receptor signaling among upregulated DEGs indicates that DHA-associated effects during UCS also involved innate immune signaling. Consistently, hepatic transcriptomic studies in Atlantic salmon exposed to environmental stress have identified coordinated regulation of heat-shock, endoplasmic-reticulum stress, oxidative-stress and metabolic pathways [28].

EU displayed a larger number of DEGs than DU, but its significant qRT-PCR changes were specific rather than uniformly metabolic or antioxidant. Downregulated DEGs were associated with fatty acid biosynthesis, steroid biosynthesis, protein export, and protein processing in the endoplasmic reticulum, and *SREBP2* was reduced. At the same time, *TNFα* and *CAT* were increased and *HSP90b* was reduced. Increased *CAT* is compatible with greater hydrogen-peroxide detoxification capacity, whereas increased *TNFα* indicates altered potential inflammatory signaling. Likewise, reduced HSP90b may reflect altered chaperone demand or impaired proteostasis [11,29].

The larger number of DEGs in EU and the diet-specific qRT-PCR findings support different associations with DHA and EPA under UCS. The present data do not demonstrate that EPA is superior to DHA in activating *PPARγ*, antioxidant defense, or stress resistance; DEG number reflects response breadth rather than physiological benefits. However, PPAR structure and function can differ among ray-finned fishes and from mammalian models [30]. Further studies are needed to investigate lipid metabolism and PPAR signaling in greater detail.

### 4.4. Shared and Distinct Responses to High-HUFA Diets Under UCS

The small overlap of 12 DEGs between DU vs. LU and EU vs. LU points to a limited common response to the two high-HUFA diets during UCS. At the pathway level, both comparisons included downregulated genes associated with fatty acid biosynthesis and steroid biosynthesis, suggesting that suppression of anabolic lipid pathways may form part of a shared hepatic adjustment to chronic stress when dietary HUFA supply is high. Interactions between dietary LC-PUFA supply and chronic environmental stress have also been reported for steroidogenic and innate immune responses in Atlantic salmon [31]. This interpretation agrees with the reduction in *HMGCR* in DU and *SREBP2* in EU, although the different qRT-PCR targets affected in each group indicate that the regulatory routes were not identical. The results also complement earlier observations that dietary HUFA level influences salmon robustness and intestinal responses under challenging conditions [2,4,7].

The diet-specific features were at least as prominent as the shared response. DU was characterized by reduced *PPARα* and *HMGCR* and enrichment of innate immune pathways among upregulated DEGs, whereas EU combined broader transcriptomic change with reduced *SREBP2* and *HSP90b* and increased *TNFα* and *CAT*. Protein-processing pathways in the endoplasmic reticulum were enriched only among downregulated genes in EU. These findings identify candidate mechanisms linking dietary fatty acid composition to hepatic stress adaptation. The significant reductions in *SREBP2* and *HSP90b* in EU further indicate that the EPA-rich diet was associated with changes in sterol regulation and proteostasis-related transcription during UCS.

### 4.5. Aquaculture Implications, Limitations, and Future Directions

From an aquaculture perspective, the absence of dietary effects on growth indicates that the low-HUFA diet was sufficient to maintain short-term growth under the tested conditions, while the molecular data reveal responses that may become relevant during longer or more severe challenges. The results do not yet justify phase-specific recommendations favoring DHA during low-stress periods or EPA during high-stress periods. A more defensible implication is that EPA:DHA composition may influence physiological and molecular responses in Atlantic salmon [32], and the present results further suggest that hepatic pathways may respond differently before and during UCS. This is consistent with evidence that low dietary EPA+DHA can reduce robustness under demanding production conditions even when routine performance measures are less sensitive [4]. Future studies should assess multiple EPA:DHA ratios and total inclusion levels [33], and integrate transcriptomics with lipidomics, enzyme activity, stress endocrinology, histopathology, and long-term welfare and performance. Such work will determine whether the distinct signatures observed here represent adaptive protection, metabolic cost, or neutral remodeling.

## 5. Conclusions

This study investigated hepatic transcriptomic responses in Atlantic salmon fed DHA- or EPA-enriched diets under unstressed and UCS conditions. Under unstressed conditions, the DHA-enriched diet elicited a broader hepatic transcriptomic response than the EPA-enriched diet, with diet-specific modulation of genes involved in lipid metabolism and redox regulation. In contrast, under UCS conditions, the EPA-enriched diet elicited a broader but distinct hepatic transcriptional response compared with the DHA-rich diet. Collectively, these findings highlight the stress-dependent effects of dietary DHA and EPA on hepatic transcriptional regulation and provide a foundation for optimizing EPA- and DHA-based nutritional strategies in Atlantic salmon production.

## Figures and Tables

**Table 1:** Ingredient formulation, oil-blend composition, and selected analyzed fatty acid composition of the experimental diets.

Item	LF	DF	EF
Ingredient formulation (g/kg of diet)			
Fish Meal SA Superprime	30	30	30
Fish Meal SA Krill	20	20	20
Sunflower expeller, low fiber	21.4	21.4	21.4
Wheat gluten	127	127	127
Maize gluten	50	50	50
Pea protein	120	120	120
Wheat	140	140	140
Oil blend	192	192	192
Vitamins, minerals and additives	35.7	35.7	35.7
Crystalline amino acids	21	21	21
Lucantin Pink, BASF	0.55	0.55	0.55
Oil-blend composition (% of oil blend)			
Rapeseed oil	92	71	66
Vevodar^®^ oil	2	0	0
Epax 1050	3	29	0
Epax 4510	3	0	34
Selected analyzed fatty acid composition			
Crude lipid (%)	20.2	19.5	19.0
EPA (g/kg of diet)	4.0	6.5	25.0
DHA (g/kg of diet)	4.5	28.7	8.1
EPA+DHA (g/kg of diet)	8.5	35.2	33.1
HUFA (% of total fatty acids)	6.63	23.08	21.57
EPA+DHA (% of total fatty acids)	4.53	19.44	18.77

Note. The diet formulation and composition data were previously reported for the same experimental feeding trial by Løvmo et al. (2021) [2] and are presented here in an adapted form for the three dietary treatments included in the present study.

**Table 2:** Primers used for qRT-PCR in this study.

Gene	Reference	Primer Name	Primer Sequences (5′-3′)
*EF1a*	BT072490.1	ss*EF1a*F ss*EF1a*R	CACCACCGGCCATCTGATCTACAA TCAGCAGCCTCCTTCTCGAACTTC
*EF1αB*	BG933897	ss*EF1αB*F ss*EF1αB*R	TGCCCCTCCAGGATGTCTAC CACGGCCCACAGGTACTG
*Beta-Actin*	AF012125	ss*bActin*F ss*bActin*R	CAACTGGGACGACATGGAGA AGTGAGCAGGACTGGGTGCT
*APOA1*	NM_001123663	ss*APOA1*F ss*APOA1*R	CTGGTCCTCGCACTAACCAT TGGACCTCTGTGCAGTCAAC
*APOA4*	BT048822	ss*APOA4*F ss*APOA4*R	CAGGACCAGTCTCAGCAACA GTTGACTTCCTGTGCCACCT
*FATP*	CA373015	ss*FATP*F ss*FATP*R	AGGAGAGAACGTCTCCACCA CGCATCACAGTCAAATGTCC
*PPARα*	XM_036945541.1	ss*PPARα*F ss*PPARα*R	GCTTCATCACCAGGGAGTTT TCACTGTCATCCAGCTCCAG
*PPARγ*	XM_036984365.1	ss*PPARγ*F ss*PPARγ*R	TGCTGCAGGCTGAGTTTATG CAGGGGAAAGTGTCTGTGGT
*HSP70*	AJ632154	ss*HSP70*F ss*HSP70*R	CATCGACTTCTACACCTCCATCAC CTGAAGAGGTCGGAACACATCTC
*HSP90b*	AF135117	ss*HSP90b*F ss*HSP90b*R	GTGTGAACAATGGGAAATGGAACA CAGCGTGCATGTTATGTTGCA
*TNFα*	AY848945.1	ss*TNFα*F ss*TNFα*R	GGCGAGCATACCACTCCTCT TCGGACTCAGCATCACCGTA
*TGFβ1/5*	EU082211.1	ss*TGFβ1/5*F ss*TGFβ1/5*R	ATCGGAGAGTTGCTGTGTGC GGGCCGATGCAGTAGTTAGC
*CAT*	NM_001140302.1	ss*CAT*F ss*CAT*R	CCAGATGTGGGCCGCTAACAA TCTGGCGCTCCTCCTCATTC
*SOD*	XM_014145196.3	ss*SOD*F ss*SOD*R	GTTTCTCTCCAGCCTGCTCTAAG CCGCTCTCCTTGTCGAAGC
*GPX*	EH033571.1	ss*GPX*F ss*GPX*R	TCTCCTGCCATAACGCTTGA GTGATGAGCCCATGGCCTTA
*GPX4b*	BT044014.1	ss*GPX4b*F ss*GPX4b*R	GGGCAGGTGGTGAAGAGATA CACGCTAGGTTTCATCAGGC
*GPX7*	NM_001140889.2	ss*GPX7*F ss*GPX7*R	TGGGAAAGTCATGGATGCCT GCTCAGGGTGTTTTGTTGCA
*GR*	XM_014199133.3	ss*GR*F ss*GR*R	CCAGTGATGGCTTTTTTGAACTT CCGGCCCCCACTATGAC
*SREBP1*	HM561860	ss*SREBP1*F ss*SREBP1*R	GCCATGCGCAGGTTGTTTCTTCA TCTGGCCAGGACGCATCTCACACT
*SREBP2*	HM561861	ss*SREBP2*F ss*SREBP2*R	TCGCGGCCTCCTGATGATT AGGGCTAGGTGACTGTTCTGG
*HMGCR*	NM_001173919	ss*HMGCR*F ss*HMGCR*R	CCTTCAGCCATGAACTGGAT TCCTGTCCACAGGCAATGTA
*FASN*	DW551395.1	ss*FASN*F ss*FASN*R	ACCGCCAAGCTCAGTGTGC CAGGCCCCAAAGGAGTAGC

**Table 3:** Clean read quality metrics of each in average.

Group	Total Clean Reads (M)	Total Clean Bases (Gb)	Q20 Clean Reads (%)	Q30 Clean Reads (%)	Clean-Read Ratio (%)	rRNA Ratio (%)
LF	43.50	4.35	98.17	94.49	92.86	0.61
DF	44.02	4.40	98.53	95.42	92.72	1.02
EF	43.98	4.40	98.61	95.63	92.63	0.68
LU	43.57	4.36	98.39	94.95	92.99	0.84
DU	43.24	4.32	98.55	95.42	93.57	2.42
EU	43.92	4.39	98.29	94.65	92.71	2.46

**Table 4:** Summary of genome mapping of each in average.

Group	Total Clean Reads	Total Mapping Ratio (%)	Uniquely Mapping Ratio (%)
LF	43,499,293	95.00%	67.69%
DF	44,019,407	94.77%	68.65%
EF	43,976,033	94.72%	68.63%
LU	43,567,517	95.05%	67.55%
DU	43,240,849	94.58%	67.66%
EU	43,916,595	93.99%	68.06%

**Table 5:** Genes and transcript statistics of each in average.

Group	Total Gene Number	Known Gene Number	Novel Gene Number	Total Transcript Number	Known Transcript Number	Novel Transcript Number
LF	34,905	31,606	3299	53,782	33,976	19,806
DF	35,842	32,438	3405	56,288	35,579	20,709
EF	35,743	32,365	3378	56,080	35,631	20,449
LU	34,589	31,341	3248	54,487	34,755	19,733
DU	33,313	30,291	3022	50,178	31,863	18,316
EU	32,039	29,180	2859	46,735	29,401	17,334

## Data Availability

The raw RNA-sequencing data generated in this study have been deposited in the NCBI Sequence Read Archive (SRA) under accession number PRJNA1458987.

## Abbreviations

The following abbreviations are used in this manuscript:

DHA	Docosahexaenoic acid
EPA	Eicosapentaenoic acid
HUFA	Highly unsaturated fatty acid
LC-PUFA	Long-chain polyunsaturated fatty acid
UCS	Unpredictable chronic stress
LF	Low-HUFA diet group
DF	DHA-rich diet group
EF	EPA-rich diet group
LU	Low-HUFA diet group after UCS
DU	DHA-rich diet group after UCS
EU	EPA-rich diet group after UCS
FA	Fatty acid
RNA-seq	RNA sequencing
DEG	Differentially expressed gene
GO	Gene Ontology
KEGG	Kyoto Encyclopedia of Genes and Genomes
qRT-PCR	Quantitative reverse transcription polymerase chain reaction
RIN	RNA integrity number
NCBI	National Center for Biotechnology Information
SRA	Sequence Read Archive
